# A Comparative Multi-Bioassay Assessment of Tetracycline Mixture Toxicity in Water and Soil Using Harmonized Dose–Response Modeling

**DOI:** 10.3390/jox16040122

**Published:** 2026-07-01

**Authors:** Chrysi A. Papadimitriou, Christina Emmanouil, Amalia Moriki, Vasileios Bartzis

**Affiliations:** 1 Laboratory of Ecology and Environmental Science, Department of Crop Science, Agricultural University of Athens, Iera Odos 75, 11855 Athens, Greece; mparbasilhs@yahoo.gr; 2 School of Spatial Planning and Development, Aristotle University of Thessaloniki, 54124 Thessaloniki, Greece; 3 Department of Food Science and Technology, International Hellenic University, 57400 Thessaloniki, Greece; moriki@ihu.gr

**Keywords:** tetracyclines, bioassays, mixture toxicity, concentration addition, emerging pollutants

## Abstract

Tetracyclines (TCs) are antibiotics widely used in human and veterinary medicine as well as in agricultural practices. They may be retained in soil or drift into freshwater, thereby exerting effects on non-target organisms and deteriorating ecological quality. In this study, tetracycline (T), oxytetracycline (OT), chlortetracycline (CT), and their binary and ternary mixtures were evaluated using a battery of bioassays including terrestrial plants, aquatic crustaceans, a ciliate protist and a bacterial species. Results showed a concentration-dependent effect for parameter immobilization in *Daphnia magna* and *Artemia salina*, seed germination in the terrestrial plants, and bioluminescence inhibition and growth inhibition in *Aliivibrio fischeri* and *Tetrahymena thermophila*, respectively. For *A. fischeri*, statistically significant interactions were observed between dose and exposure time. *A. salina* demonstrated greater sensitivity than *D. magna* in all cases. Both *A. salina* and *A. fischeri* showed increased toxicity to OT and the ternary mixture. Dicots presented greater sensitivity than the monocot species in all cases. In the combined exposures, there was a deviation from the concentration addition (CA) model, with possible synergism for CT + T and the ternary mixture for *A. fischeri*. The concurrent environmental exposure of non-target organisms to TCs should be investigated further.

## 1. Introduction

Pharmaceuticals and personal care products are being exponentially detected in a variety of hydrological, climatic, and land-use settings across the world [1]. Of these pharmaceuticals, tetracyclines (TCs) rank among the most widely used antibiotics in human medicine, livestock production and aquaculture [2,3,4,5,6]. Global consumption of TCs in food-producing animals amounted to 33,305 tons and is expected to increase by 9% by 2030 [7]. TCs are employed for both therapeutic and preventive treatment of animal diseases and have historically been utilized as growth promoters to improve feed efficiency in livestock [3,8,9]. Livestock farming and aquaculture are primary areas of TC use, where antibiotics are administered to promote growth. Xu et al. [5] reported that 180,000 tons of raw antibiotics were used annually in China for human and agricultural purposes, equating to 138 g per capita. In animal husbandry, 172 mg, 148 mg, and 45 mg of antibiotics/kg were administered to slaughtered pigs, chickens, and cows, respectively. Approximately 70–90% of TCs are excreted as unmetabolized parent compounds or metabolites after consumption by humans and animals due to incomplete absorption and metabolism [10] and accumulate in manures [11], soils [3,8], sediments, and surface and groundwaters [12], raising concerns about ecotoxicity, food-chain transfer, and antimicrobial resistance. The latter is a well-known threat for public health according to ECDC, and it is accelerated by the extensive use of antibiotics, which exerts an ecological pressure on microorganisms and contributes to the emergence and selection of antimicrobial-resistant counterparts [13]. Pharmaceutical industry waste represents another source of tetracycline contamination [4,14]. Previous studies identified the low degradability of tetracycline as an ecological concern. TCs and their byproducts can migrate from soil to aquatic environments via surface runoff and infiltration [15].

**Figure 1:**
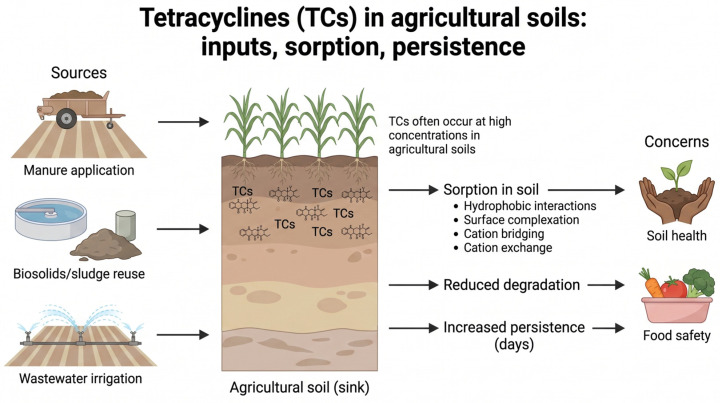
TCs and related compounds in agricultural soils.

Their sorption in soil occurs via hydrophobic interactions, surface complexation, cation bridging, and cation exchange [16,17], reducing degradation and increasing persistence in soils [18,19]. TCs exhibit variable persistence depending on environmental conditions; while they may degrade within days under certain conditions, strong sorption to soils and continuous inputs can result in their long-term environmental presence [8,17,18]. The widespread occurrence of TCs in agricultural soils has been documented in research articles [9,20] and review studies [21,22]. Additionally, their sorption and dissipation kinetics have been extensively explored [23,24,25,26,27] (Figure 1).

Toxic effects have been documented for aquatic primary producers, invertebrates, and fish, as well as for plants and soil microorganisms, with algae and cyanobacteria being among the most sensitive groups [3,4,5,28,29,30,31,32].

Despite this growing body of evidence of environmental concern, monitoring of TCs remains fragmented: data are geographically uneven, often focus on wastewater treatment plants, and rarely integrate aquatic and terrestrial compartments or track toxic transformation products and resistance determinants [3,5,6,20,29,33,34]. Several reviews highlight the absence of robust discharge limits for TCs, the lack of systematic environmental databases, and the lag between laboratory evidence and regulatory implementation [3,4,6,30,34]; for the substances under examination (T and OT), they have just now started receiving widespread attention due to their inclusion in the EU Surface Water Watch List [35].

**Figure 2:**
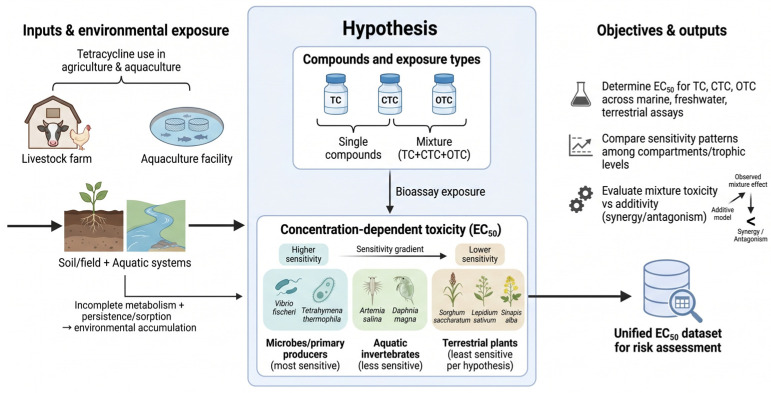
Overview of the research hypothesis tested.

To address these gaps, the present study hypothesizes that tetracycline, chlortetracycline, and oxytetracycline—individually and in mixtures—exhibit concentration-dependent toxicity that varies across trophic levels and that mixture effects can be quantified against additive models to reveal potential synergistic or antagonistic interactions. Accordingly, the objectives are to (i) determine EC_50_ values for each compound across a battery of marine, freshwater, and terrestrial bioassays (*Aliivibrio fischeri* (previously *Vibrio fischeri*), *Tetrahymena thermophila*, *Artemia salina*, *Daphnia magna*, *Sorghum saccharatum*, *Lepidium sativum*, *Sinapis alba*), (ii) compare sensitivity patterns among organisms representing distinct environmental compartments, and (iii) evaluate mixture toxicity using harmonized dose–response modeling, thereby generating a unified dataset that can inform risk assessment and support the development of discharge limits currently lacking for tetracyclines in agricultural settings (Figure 2).

The selection of tetracycline (T), oxytetracycline (OT), and chlortetracycline (CT) was based on their widespread use in veterinary medicine and animal production, their frequent detection in agricultural matrices, and their strong relevance to soil contamination through manure application, slurry disposal, and runoff from farming systems. These compounds are among the most representative tetracyclines associated with agricultural contamination and are therefore appropriate model substances for comparative ecotoxicological assessment. Their selection is also aligned with current European priorities on soil health, soil monitoring, and protection of the soil–water interface, which increasingly recognize the importance of contaminants introduced by agricultural practices, including veterinary pharmaceuticals.

Most ecotoxicological studies on tetracyclines have focused primarily on aquatic organisms and waterborne exposure, whereas fewer studies have integrated terrestrial and aquatic compartments within a common experimental framework. To address this gap, the present study included both single compounds and environmentally relevant binary and ternary mixtures and applied a test battery covering microbial, aquatic, and terrestrial organisms. This design enabled the comparison of sensitivity patterns across trophic levels and compartments and provided a broader perspective on tetracycline toxicity in agricultural environments.

## 2. Materials and Methods

### 2.1. Substances and Mixtures

Analytical-grade tetracycline (T), oxytetracycline (OT), and chlortetracycline (CT) (≥98% purity, Techline, Oinofyta, Greece were used to prepare stock solutions. All solutions were prepared using deionized water under controlled laboratory conditions. Because tetracyclines are known to be chemically unstable, all working solutions were freshly prepared immediately prior to testing, and the initial pH of the exposure solutions was monitored, ranging from 6.4 to 7.8. This near-neutral range was considered suitable for maintaining standardized assay conditions while avoiding extreme pH values that could markedly alter tetracycline speciation. However, the aim of the study was comparative ecotoxicological assessment rather than analytical evaluation of antibiotic transformation. Accordingly, no dedicated photodegradation-control treatment and no time-resolved chemical confirmation of tetracycline stability were included in order to assimilate the possible environmental fate.

The study was conducted under standardized experimental conditions and does not involve environmental sampling, aiming instead to establish controlled dose–response relationships. The concentration ranges selected in this study exceed typical environmental levels and were chosen to establish clear dose–response relationships and derive EC_50_ values in accordance with standardized ecotoxicological protocols (OECD/ISO) [36,37,38]. Therefore, the results should be interpreted primarily in terms of hazard identification rather than direct environmental exposure. Fixed mixture ratios (1:1, 7:3, and 3.5:3.5:3) were selected to enable the evaluation of potential interactions under controlled conditions. The concentration ranges selected in this study exceed those typically reported for ambient environmental waters and soils. This choice was deliberate because the objective was not to simulate average field exposure but to establish clear concentration–response relationships, derive EC_50_ values, and compare the relative sensitivity of species and mixtures under standardized laboratory conditions. Such an approach is consistent with acute ecotoxicological hazard identification, where sufficiently broad concentration ranges are required to detect measurable effects across organisms with different sensitivities and to support robust statistical modeling. In addition, tetracyclines are not restricted to diffuse low-level contamination but may occur at substantially elevated concentrations in point-source contamination settings worldwide, including pharmaceutical manufacturing effluents, wastewater treatment plant influents, livestock manure and slurry, aquaculture-related discharges, and manure-amended soils and runoff-affected [3,4,5,11,12,25,26,39,40,41]. Therefore, the concentrations applied here should be interpreted primarily as suitable for hazard characterization and comparative mixture assessment rather than as direct proxies of typical environmental exposure.

### 2.2. Battery of Tests

#### 2.2.1. *A. fischeri*—Microtox Bioassay

The bioluminescence bacteria *A. fischeri* were obtained in freeze-dried form (SDI, Itasca, IL, USA) and activated prior to use by a reconstitution solution. Since *A. fischeri* is a marine organism, adjustment of the osmotic pressure up to 2% salinity using a concentrated salt solution was applied (solution containing 22% NaCl in deionized water). The light emitted from the test organisms, obtained by their direct contact with the samples, was measured using the Microtox 500 analyzer (SDI) within a short exposure time of 15 min. All treatments and controls were tested in triplicate. Data processing was performed using Microtox Omni software 4.2 (SDI), according to the Microtox 82% Basic Test that follows the ISO protocol 11348-3 ISO [36].

#### 2.2.2. *D. magna* and *A. salina* Bioassays

For determination of the toxic effects on *D. magna*, the Daphtoxkit F (Microbiotests, Ghent, Belgium) was used. The organisms were obtained in the form of ephippia and were hatched for 3–4 days in an incubator under continuous illumination of 6000 lux at 22 °C. Once the organisms were hatched, five organisms (×4 replicates) were transferred to plastic test wells, containing the control medium and the samples. The toxic effect was evaluated as the percentage of non-viable/immobilized organisms after 24 h of exposure to the samples in the absence of light. Similarly, for the determination of toxic effects on *A. salina*, the Artoxkit F by Microbiotests for short-term acute mortality (24 h exposure static test) was used after the appropriate hatching of the cysts. All treatments and controls were tested in triplicate. Both *D. magna* and *A. salina* protocols were based on OECD and ISO guidelines, respectively [37,38].

#### 2.2.3. *T. thermophila* Bioassay

In the bioassay with the ciliate protozoan *T. thermophila*, the growth inhibition of the ciliate protozoan was evaluated, following the manufacturer’s standardized 24 h protocol F (Microbiotests, Ghent, Belgium). The test is based on the optical density measurement of the food substrate provided to the ciliates in 1 cm disposable spectrophotometric cells. All treatments and controls were tested in triplicate.

#### 2.2.4. Terrestrial Plants Phytotoxicity Bioassay

Samples were tested for their phytotoxic properties using three plant species, *S. alba*, *S. saccharatum*, and *L. sativum* (Phytotoxkit, MicroBioTests Inc., Ghent, Belgium), according to [37]. The control substrate used in this bioassay was the reference soil supplied with the Phytotoxkit, which is a modified OECD artificial soil composed of 85% sand, 10% kaolin, and 5% peat, with pH adjusted by calcium carbonate. According to the manufacturer’s protocol, 35 mL of water was added to 90 cm^3^ of reference soil to obtain 100% water saturation at the start of the test. Salinity was not provided separately in the kit specifications. Ten seeds from each species were placed in flat, shallow transparent test plates composed of two compartments, the lower one containing the reference soil saturated to its water holding capacity. All samples were prepared in triplicate, and the average values are presented in the results. The test plates with the seeds were incubated for 3 d at 25 °C. The inhibition of seed germination and of root growth was calculated by Equation (1):

(1) $$I=\frac{A\;-\;B}{A}\;\times 100$$

where I is the percentage of inhibition of seed germination or root growth, *A* is the mean seed germination or root length for the control soil, and *B* is the mean seed germination or root length for the examined substances/mixtures.

#### 2.2.5. Statistical Analysis

The effect of concentration as well as the effect of exposure time on *A. fischeri* inhibition was analyzed using repeated measures ANOVA GLM, with % inhibition as the response variable and dose and exposure time as independent variables. The test was applied for the single substance exposures (CT, T, OT) as well as for the binary and the ternary mixtures. Model fit was evaluated using Mauchly’s test of sphericity. The relationship between concentration and biological response for *T. thermophila* was evaluated using linear regression analysis. Percentage inhibition was used as the dependent variable and log-transformed concentration as the predictor.

The effect of concentration on *D. magna* immobilization was analyzed using a generalized linear model (GENLIM), with the number of immobilized organisms as the response variable and the total number of exposed organisms as the binomial denominator. A binomial distribution with a logit link function was applied, based on log-transformed concentrations. Model fit was evaluated using deviance and Pearson chi-square statistics. The same procedure was performed for *A. salina* immobilization. The test was applied for the single substance exposure (CT, T, OT) as well as for the binary and the ternary mixtures. The effect of concentration on seed germination was also analyzed through a generalized linear model (GENLIM), with the number of non-germinating seeds as the response variable and the total number of seeds (10 per replicate) as the binomial denominator. Plant species were included as a fixed factor and log-transformed concentration as a continuous covariate, and the species × concentration interaction was also assessed. Statistics were performed only for those compounds that exhibited an apparent dose–response interrelation (i.e., when germination inhibition was initially non-existent and then had an abrupt increase at the highest concentration tested, no statistical analysis was applied). Consequently, for the experiments of the present study, comparisons were made only for CT and the ternary mixture. All analyses were performed by SPSS28 (IBM, Armonk, NY, USA).

### 2.3. Determination of EC_50_ Values Using Probit Analysis

Median effective concentrations (EC_50_) were estimated using probit analysis, a standard approach for modeling quantal or proportion-type dose–response data in ecotoxicology. In this approach, the proportion of affected organisms or replicates at each test concentration is transformed to a probit scale and related to the logarithm of concentration, assuming that tolerance within the tested population follows an approximately normal distribution after transformation.

For each test concentration, the response was first expressed as the proportion of affected units relative to the total number of exposed units, according to the specific biological endpoint of each assay (e.g., immobilization, inhibition of germination, or growth inhibition). When required, responses were corrected on the basis of control performance. To avoid undefined probit values for proportions equal to 0 or 1, a continuity correction was applied prior to transformation:

(2) $$p=\frac{r+0.5}{n+1}$$

where *r* is the number of affected units and *n* is the total number of exposed units. The tested concentrations were then transformed to natural logarithms, and the corrected response proportions were converted to probit units. A linear regression model was fitted according to the following general equation:

(3) $$Y=ax\ln\left(C\right)+b$$

where Y is the probit-transformed response, a is the intercept, b is the slope of the concentration–response relationship, and C is the concentration. In this model, the slope parameter (b) describes the steepness of the dose–response curve, with higher values indicating a more abrupt change in effect over a narrower concentration range.

The EC_50_ corresponds to the concentration expected to produce a 50% effect. Under the probit transformation used here, this value is obtained from the fitted regression by setting the transformed response to the probit equivalent of the 50% effect and solving for concentration:

(4) $${EC}_{50}=e^{-\frac{b}{a}}$$

where a is the intercept and b is the slope coefficient of the fitted model. EC_50_ values were then back-transformed and reported in the original concentration units.

Model performance was evaluated using the coefficient of determination (R^2^) together with visual inspection of the fitted concentration–response relationship. In cases where the experimental data did not reach the 50% effect within the tested concentration range, EC_50_ values were extrapolated from the fitted model.

### 2.4. Modeling the Mixture Toxicity

When multiple chemicals act on an organism, their combined effect can be estimated using predictive models. This is especially useful for toxicity effects. In the present study, the concentration addition (CA) model was applied as a screening-level predictive approach. The CA model assumes that mixture components act similarly and contribute to the overall effect according to their relative proportions in the mixture. Under this assumption, one component may be considered a partial substitute for another, and the combined effect can be predicted from the toxicity of the individual components. The model in its simple form does not account for environmental variability, transformation products, or bioaccumulation.

The formula that visualizes the above is the following:

(5) $$IC_{x}mix=1/\sum_{i=1}^{n}\frac{p_{i}}{\begin{array}{c} IC_{xi} \end{array}}$$

with *IC_x_mix*: total concentration of the mixture that causes *x* effect; *p_i_*: the proportion of component *i* in the mixture; *ICxi:* the concentration of component *i* that would cause x effect [39].

The experimentally determined IC_50_ values were subsequently compared with those predicted by the CA model (Formula (5)). Agreement between observed and predicted values indicates additive behavior, whereas substantial deviations suggest non-additive interactions. Specifically, observed IC_50_ values markedly lower than those predicted indicate synergistic interactions, while higher values indicate antagonistic interactions. Following common ecotoxicological practice, deviations greater than a factor of two were considered indicative of departure from CA. Calculations were performed for all tested mixtures for effects on *A. fischeri*, *D. magna* and *A. salina*.

## 3. Results

The data are shown in Supplementary Material S1.

### 3.1. Effects on A. fischeri

**Figure 3:**
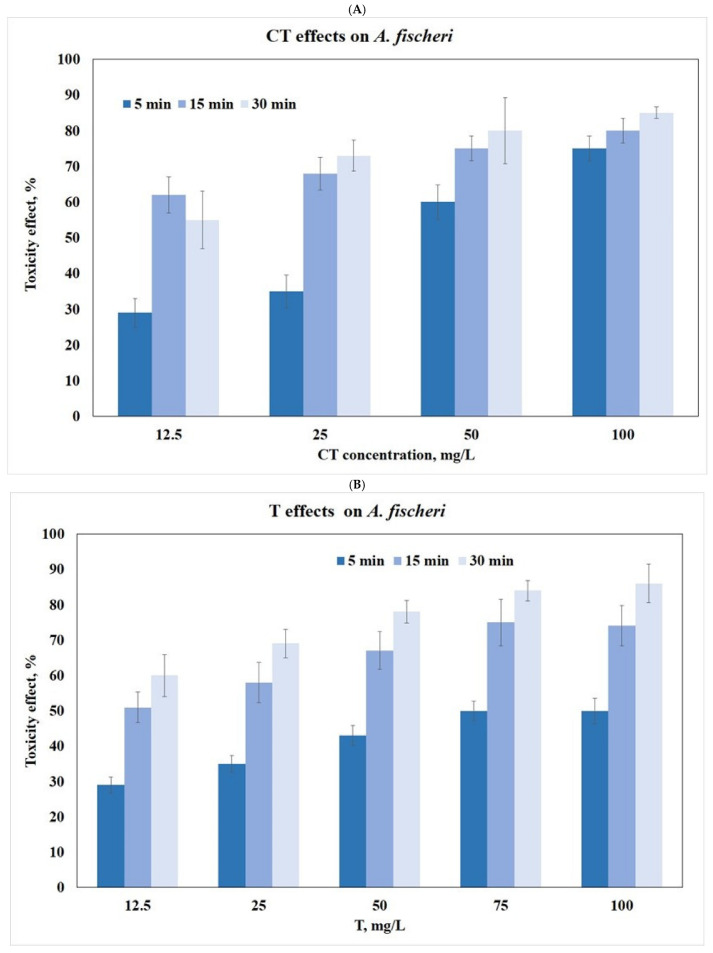

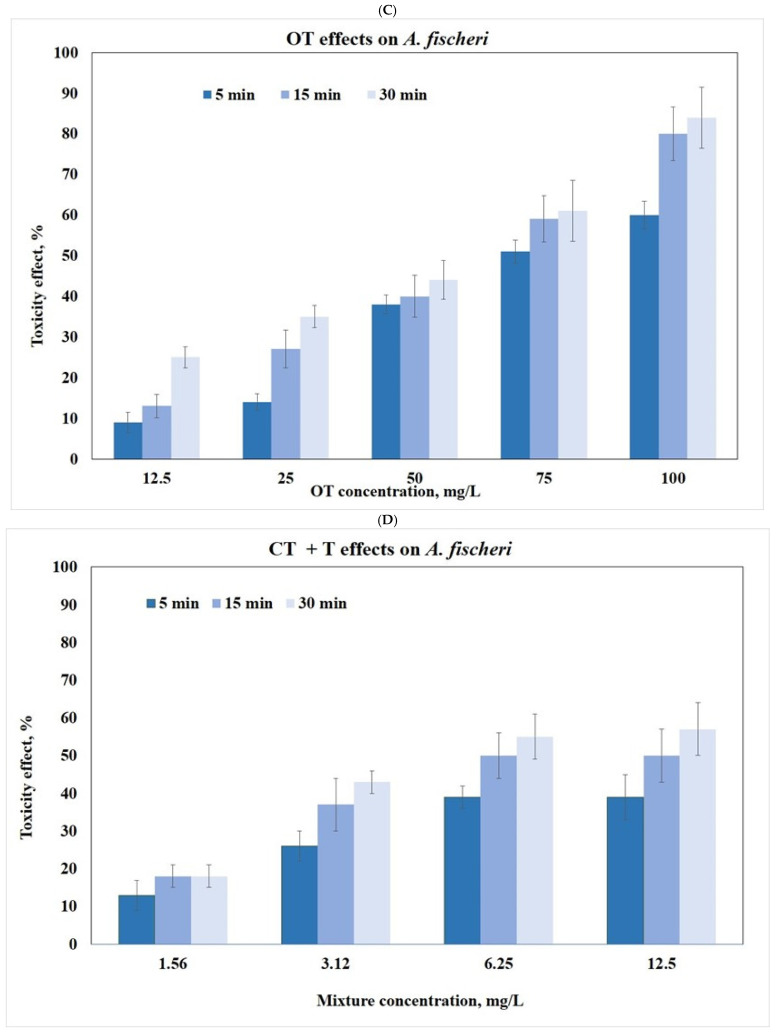

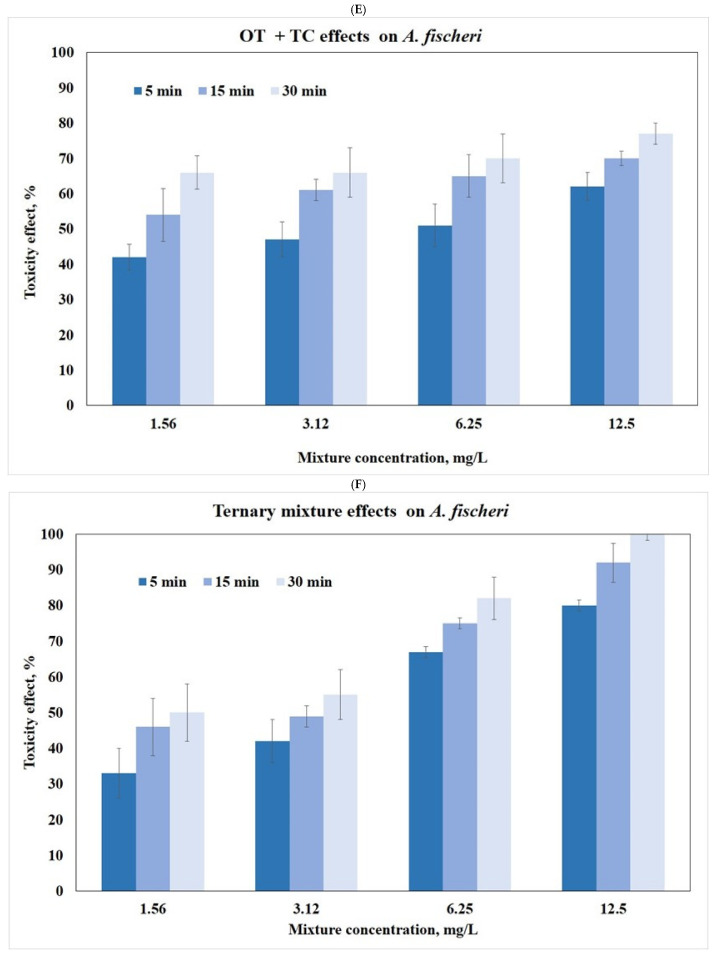
Effects of single TCs and their mixtures on *A. fischeri*. (**A**): CT effects; (**B**): T effects; (**C**): OT effects; (**D**): CT + T effects; (**E**): OT + T effects; (**F**): tertiary mixture effects. The dose–response model for each TC combination was evaluated using repeated measures ANOVA GLM, with % inhibition as the response variable and dose and exposure time as explanatory variables.

For the single exposure of *A. fischeri* to CT, data were appropriate according to Mauchly’s test of sphericity. There was a significant effect of time duration and of dose to the toxicological response (% inhibition). There was also a significant time × dose interaction. For the single exposure of *A. fischeri* to T, data were appropriate according to Mauchly’s test of sphericity. There was a significant effect of time duration and of dose to the toxicological response (% inhibition), but the dose–response relationship was not as prominent as before. There was no significant time x dose interaction. For the single exposure to OT, there was a violation of Mauchly’s test of sphericity; thus, the adjusted results (Greenhouse–Geisser) were used. There was a significant effect of time duration and of dose to the toxicological response (% inhibition). There was no significant time x dose interaction. For the binary CT + T mixture, there was a highly significant effect of time on inhibition. There was a significant time × dose interaction as well as a concentration-dependent inhibition. However, for the binary OT + T mixture, there was no significant time × dose interaction. All statistical comparisons are shown in Table 1, while the toxicity effects are presented in Figure 3.

### 3.2. Effects on T. thermophila

**Figure 4:**
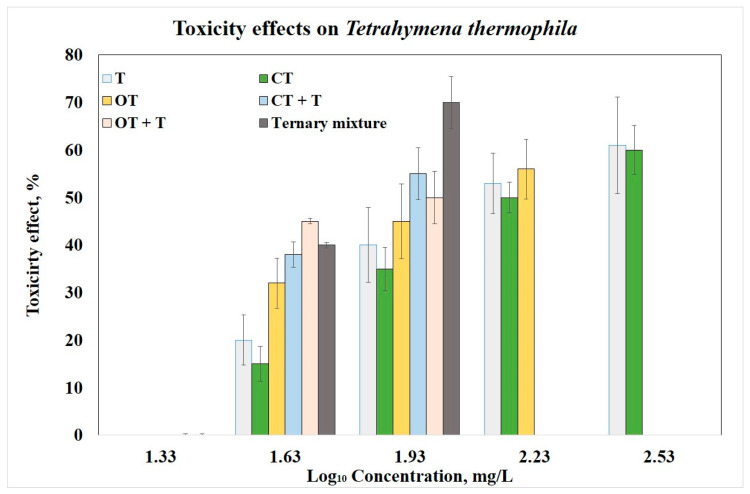
Effects of single TCs and their mixtures on *T. thermophila.* The dose–response model for each TC combination was evaluated using linear regression, with % inhibition as the response variable and dose as the predictor variables.

For the single exposure of *T. thermophila* to CT, the linear regression model explained 77.6% of the variance, with a significant positive relationship between log-transformed concentrations and percentage inhibition (F = 51.71, *p* < 0.001). For the single exposure of *T. thermophila* to T, the linear regression model explained 80.2% of the variance, with a significant positive relationship between log-transformed concentrations and percentage inhibition (F = 57.88, *p* < 0.001). Finally, the linear regression model for the single exposure to OT explained 89.2% of the variance, with a significant positive relationship between log-transformed concentrations and percentage inhibition (F = 91.58, *p* < 0.001). The model for the binary mixture of CT + T explained 89.8% of the variance, with a significant positive relationship between log-transformed concentrations and percentage inhibition (F = 71.45, *p* < 0.001). In a similar way, the parameters for the OT + T mixture were adjusted (R^2^ = 0.76, F = 27.23, *p* < 0.01). The mixture of all three substances explained 85.1% of the variance in the model, with a significant positive relationship between dose and response (F = 46.68, *p* < 0.001). The results are shown in Figure 4.

### 3.3. Effects on D. magna and A. salina

**Figure 5:**
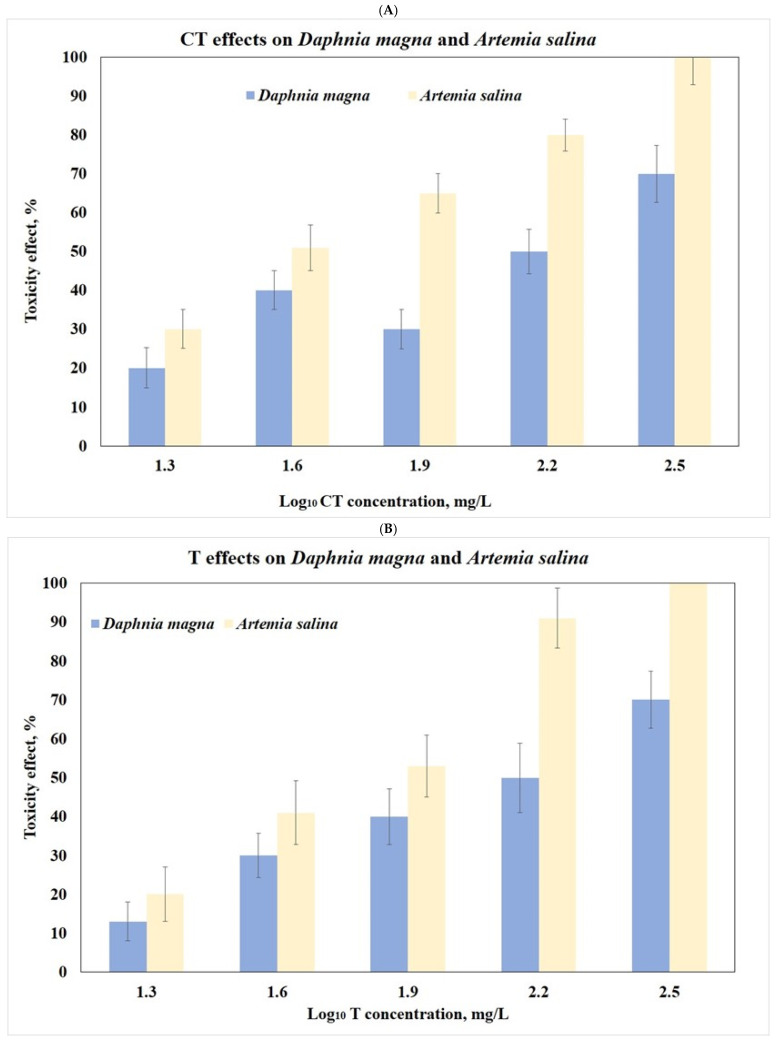

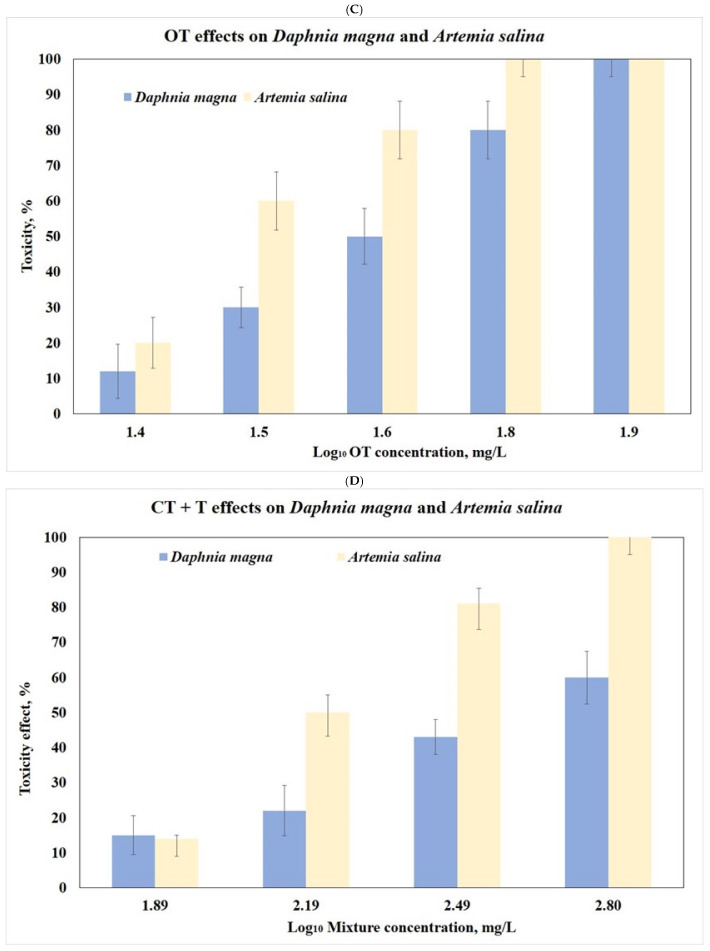

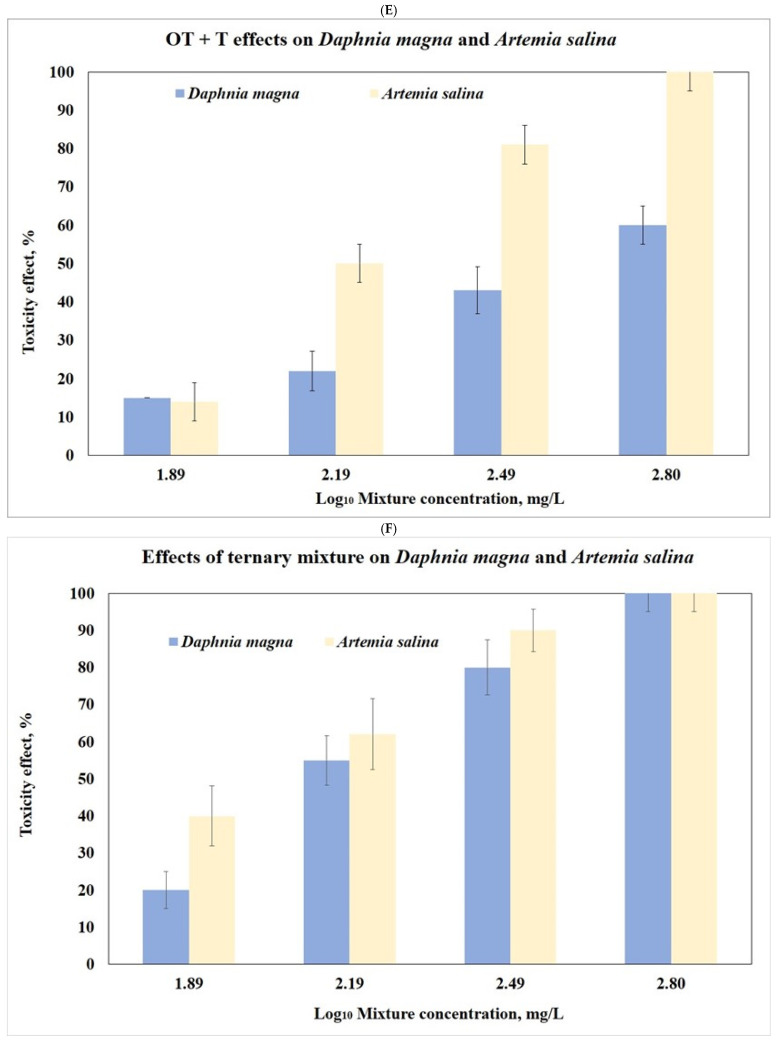
Effects of single TCs and their mixtures on *D. magna* and *A. salina.* (**A**): CT effects; (**B**): T effects; (**C**): OT effects; (**D**): CT + T effects; (**E**): OT + T effects; (**F**): ternary mixture effects. The dose–response model for each TC combination was evaluated using a generalized linear model (GENLIM), with the number of immobilized organisms as the response variable and log-transformed dose as the predictor variable.

The present results demonstrate a clear concentration–response relationship for tetracyclines (TCs), with toxicity increasing progressively with rising log_10_-transformed concentrations for both *D. magna* and *A. salina* (Figure 5). This pattern is consistent with the established ecotoxicological behavior of antibiotics in aquatic systems, where dose-dependent effects reflect increasing bioavailability and interaction with physiological targets [42].

For the single exposure of *D. magna* to CT, model fit was deemed good. There was a significant effect of dose on the toxicological response (% inhibition); as such, the results confirm a clear dose-dependent toxic effect. For the single exposure of *D. magna* to T, model fit was deemed good. There was again a significant effect of dose on the toxicological response (% inhibition) (*p* < 0.001). For the single exposure of *D. magna* to OT, model fit was deemed average because the relevant parameters were >1. There was a significant effect of dose on the toxicological response (% inhibition) (*p* < 0.001). The binary (CT + T or OT + T) and ternary mixtures produced dose–response results. Model fits were good or adequate. Dose–response intensity was the highest in the OT + T mixture.

For the single exposure of *A. salina* to CT, model fit was deemed adequate. There was a significant effect of dose on the toxicological response (% inhibition) at the level of *p* < 0.001. Results of exposure of the organism to T followed a model with adequate fit; there was a dose–response increase in the toxicological response (*p* < 0.001). Similar results to T were shown for OT for *A. salina* (*p* < 0.001). The binary (CT + T or OT + T) and tertiary mixtures produced dose–response results. Model fits were good or adequate. Dose–response intensity was the highest in the CT + T mixture. All statistical comparisons are shown in Table 2.

### 3.4. Effects on Terrestrial Plants

**Figure 6:**
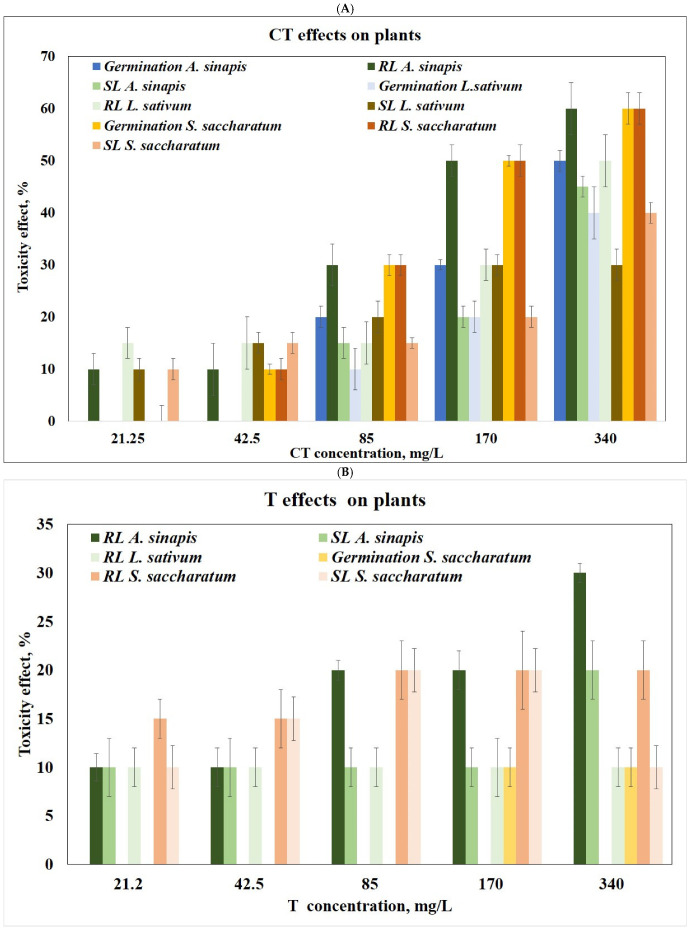

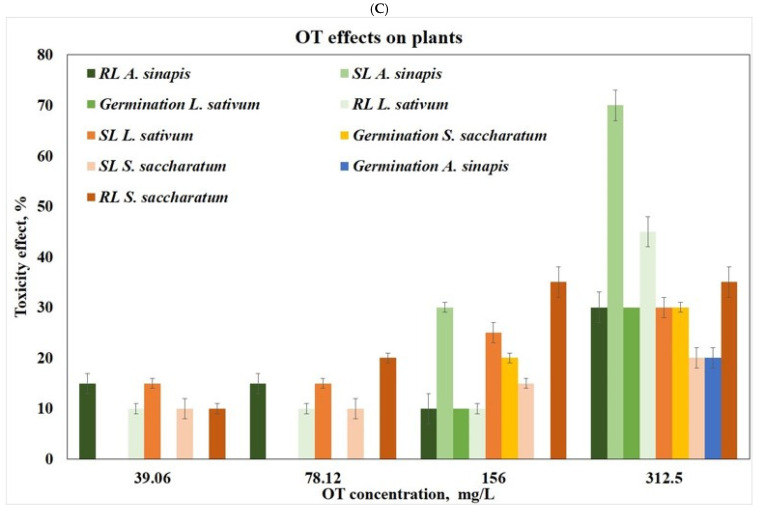
Effects of single TCs and their mixtures on *A. sinapis*, *S. saccharatum* and *L. sativum* (RL: Root Length, SL: Stem Length). (**A**): CT effects; (**B**): T effects; (**C**): OT effects. The dose–response model for CT was evaluated using a generalized linear model (GENLIM), with the number of non-germinating seeds as the response variable, plant species as a fixed factor, and concentration as a continuous covariate.

The single compounds and the binary and ternary mixtures showed varied responses for the parameters % seed germination inhibition, % root length (in relation to control) and % shoot length (in relation to control). Results for the single-compound exposure are shown in Figure 6. In general, dicots exhibited higher sensitivity. Nevertheless, a dose–response relationship was clear only in the case of CT (Likelihood Ratio χ^2^ = 90.754, *p* < 0.001). In contrast, neither species (Likelihood Ratio χ^2^ = 1.250, *p* = 0.535) nor the interaction between species and log-transformed concentration (Likelihood Ratio χ^2^ = 0.381, *p* = 0.827) were significant. However, for the ternary mixture, there was a dose–response interaction for the effects on seed germination (Likelihood Ratio χ^2^ = 18.47, *p* < 0.001), and species exhibited variable responses (Likelihood Ratio χ^2^ = 47.10, *p* < 0.001). There was no interaction between species response and dose–response interaction (Likelihood Ratio χ^2^ = 1.84, *p* >0.05). Results are shown in Figure 6.

### 3.5. EC_50_ Values for Single Substances and Mixtures and Deviation from CA

In natural environments, antibiotics rarely occur as single compounds but rather as complex mixtures resulting from agricultural runoff, manure application, and wastewater discharge [42]. As such, it is very important to predict the possible interactions between the co-existing chemicals. The results of the experimental studies in relation to the putative effects as implied by CA for the same mixtures are shown in Table 3. As a rule of thumb, only significant deviations (ratios of theoretical CA/experimental EC_50_s) of more than 2 or less than 0.5 were considered indicative of divergence of additivity. In the *A. fischeri* experimental model, the CT + T and CT + T + OT exposure caused higher inhibition than the simple sum of single effects; as such, a synergistic effect cannot be ruled out. However, an additive effect was the most probable explanation for the EC_50_ recorded for the OT + T exposure. On the contrary, exposure to CT + T caused lower toxic effects than those theoretically expected for the models of *D. magna* and *A. salina*. For the latter, all the binary and ternary combinations also produced lower-than-expected toxic effects. For the rest of the experimental exposures, there was no clear deviation from the CA mode of action.

### 3.6. Comparative Sensitivity Across Bioassays

**Figure 7:**
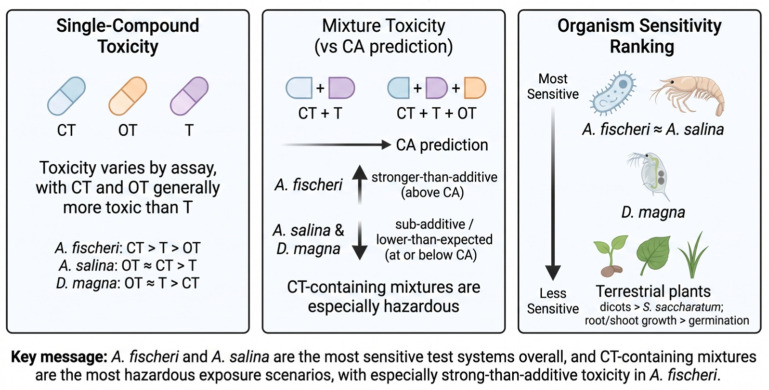
Comparative sensitivity of the bioassays to tetracyclines.

Organisms showed variable sensitivity in the different exposure scenarios. Based on the EC_50_ values and concentration–response patterns, *A. fischeri* and *A. salina* were overall the most sensitive organisms, whereas *D. magna* was less sensitive and terrestrial plants showed endpoint- and species-specific responses. Among the single compounds, chlortetracycline (CT) and oxytetracycline (OT) generally exerted stronger toxic effects than tetracycline (T), although this ranking was not identical in all assays. In *A. fischeri*, CT showed the highest single-compound toxicity, followed by T and OT, while in *A. salina*, OT and CT were more toxic than T. In *D. magna*, OT and T showed slightly higher toxicity than CT. For terrestrial plants, dicotyledonous species were generally more sensitive than *S. saccharatum*, with root and shoot growth being more responsive endpoints than germination. Regarding mixtures, the CT + T and CT + T + OT combinations were the most toxic for *A. fischeri*, showing stronger-than-additive effects compared with the CA prediction, whereas in *A. salina* and partly in *D. magna*, the mixtures tended to produce lower-than-expected toxicity. Overall, these results indicate that microbial and marine crustacean bioassays were the most responsive tools for detecting tetracycline mixture toxicity, and that CT-containing mixtures were among the most hazardous exposure scenarios tested (Figure 7).

## 4. Discussion

### 4.1. Species-Specific Sensitivity and Ecological Relevance

Although environmental concentrations are typically in ng–μg/L ranges, acute toxicity tests require higher concentrations to define thresholds. These results should not be interpreted as a direct environmental risk but as hazard identification. In the present study, TCs and their mixtures were assessed for their toxic effects on a battery of bioassays. However, in realistic scenarios, TCs’ fate is more complex, with limited catabolism in wastewater treatment plants and significant adsorption onto sewage [43]. Also, some of their degradation products, such as α-apo-OTC and β-apo-OTC, exhibit greater mobility than the parent compounds and are therefore more likely to be detected in aquatic matrices [40]. In this context, investigation into their possible ecotoxicity is paramount.

**Figure 8:**
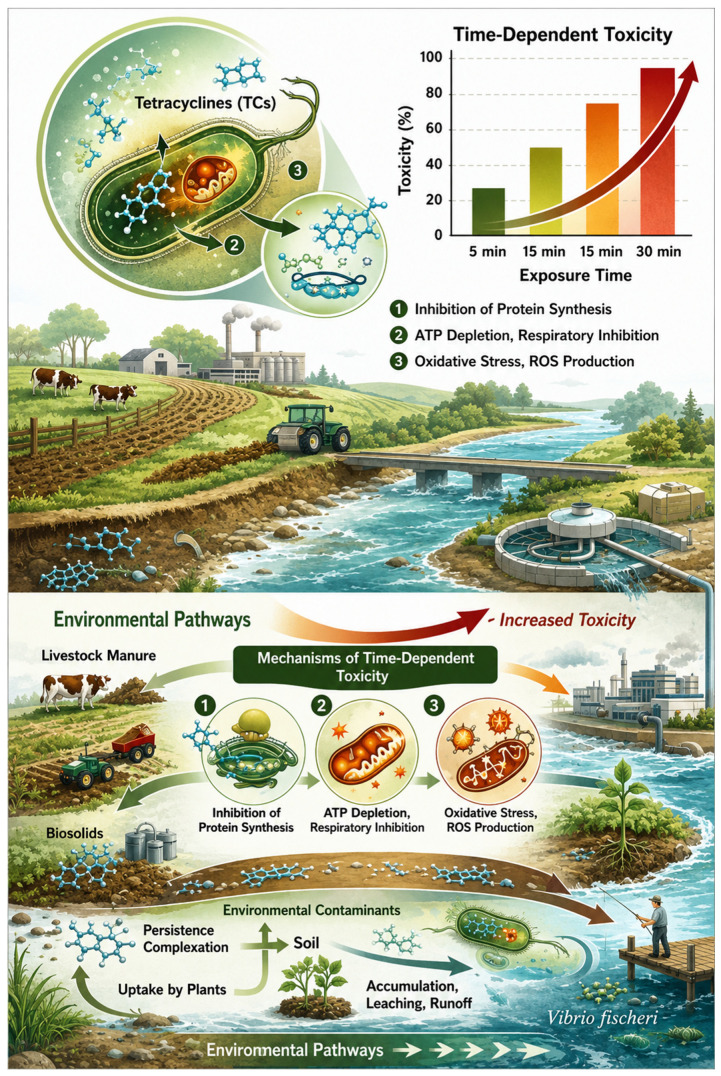
Mechanisms of time-dependent toxicity in *A. fischeri*.

The results obtained for *A. fischeri* demonstrate a clear concentration- and time-dependent increase in the toxicity of TCs. Luminescent bacterial assays in general constitute one of the most widely applied standardized microbial toxicity tests for aquatic environments [44]. Here, there was strong bioluminescence inhibition affected both by increasing concentration (12.5–100 mg L^−1^) and exposure time (5–30 min). This pattern is consistent with the fundamental principle of time-dependent toxicity, where the magnitude of toxic effects evolves over exposure time due to progressive interaction between the toxicant and biological systems [45]. In the present case, the systematic increase in inhibition from 5 to 30 min suggests that TC toxicity is not instantaneous but rather develops as intracellular processes are progressively disrupted (Figure 8). This response pattern aligns well with previous findings indicating that antibiotics, including TCs, primarily exert their effects through delayed mechanisms; by inhibiting translation at the ribosomal level, they progressively suppress bacterial growth, reproduction, and metabolic [46,47] (Figure 8). In *A. fischeri*, toxicity is measured via inhibition of bioluminescence, which is directly linked to cellular respiration and enzymatic activity. Therefore, the increasing toxicity observed over time likely reflects the gradual impairment of metabolic pathways [44]. Studies have shown that compounds with delayed modes of action exhibit increasing toxicity over time, in contrast to fast-acting toxicants that produce immediate effects [47]. Unlike conventional toxicants such as heavy metals, pharmaceuticals often target specific biochemical pathways, leading to progressive toxicity as exposure time increases [46]. This explains why short-term assays (e.g., 5 min) may underestimate the ecological risk of antibiotics, while longer exposure periods (15–30 min or beyond) reveal significantly higher toxic responses. Indeed, standardized *A. fischeri* assays such as the Microtox^®^ bioassay, which was used in the present study, explicitly include multiple exposure times to capture this temporal dimension of toxicity [45]. Furthermore, the observed inhibition of *A. fischeri* may provide an early indication of potential impacts on other aquatic organisms [44].

Regarding the experimental model organism *Tetrahymena* sp., this is a useful tool for freshwater environments. This organism is one of the most sensitive organisms among toxicological models, and it can therefore be applied as a toxicant detection probe [48]. In the present study, all single compounds and their mixtures exerted a dose-dependent effect on *T. thermophila*. In the relevant literature, this organism exhibited low sensitivity (endpoint: growth inhibition) to antibiotics such as sulfamethoxazole and lincomycin [49], although very limited information on *Tetrahymena* sp. and antibiotics is available [50]. Nevertheless, TCs can impair mitochondrial function in a variety of eukaryotic models [51], and this may also be the mode of toxicity for *Tetrahymena* sp.

Regarding crustaceans, there was a consistently higher sensitivity of *A. salina* than of *D. magna*, which may be linked to differences in environmental conditions (e.g., salinity) and organism-specific uptake and detoxification mechanisms. TC speciation and bioavailability are strongly influenced by ionic strength and pH [52], while higher salinity has been shown to potentially enhance their bioavailability through slower removal [53] and slower colloidal-assisted photochemical transformation [54]. On the other hand, *D. magna* typically exhibits moderate sensitivity [55]. In this context, the toxic effects observed in *D. magna* and *A. salina* are of particular relevance to environmental and ecological quality, as these organisms represent key components of aquatic food webs. The higher sensitivity of *Artemia salina* suggests that the ecological risk may be amplified in estuarine and coastal receiving environments, where agricultural discharges often accumulate.

**Figure 9:**
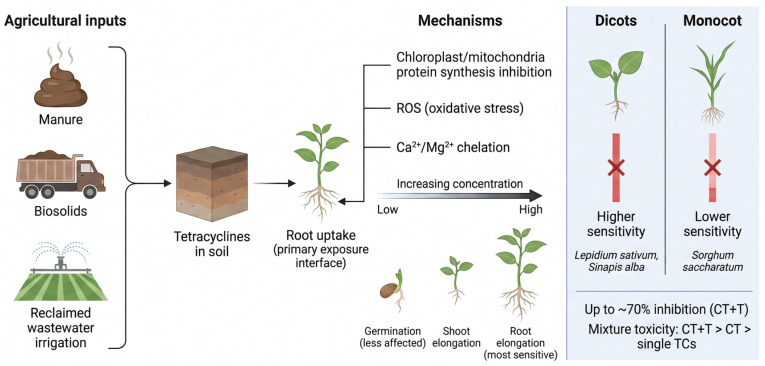
Phytotoxicity of TCs.

Regarding terrestrial plants, TCs have been phytotoxic to many species, affecting growth and germination and causing genotoxic effects [2]. TCs can easily translocate through the xylem from the root to the aerial plants; it has also been shown that they inhibit chloroplast synthase activity and may elicit antibiotic resistance in the plant rhizosphere [56]. The present results demonstrate that tetracyclines (TCs), both as single compounds and in mixtures (CT and CT + T), exert clear concentration-dependent phytotoxic effects on both monocotyledonous (*S. saccharatum*) and dicotyledonous species (*L. sativum* and *S. alba*), with marked differences in sensitivity and endpoint response. Overall, dicots exhibited greater susceptibility than the monocot species, particularly in root elongation (RL) and shoot elongation (SL), with *A. sinapis* showing the highest inhibition (up to ~70% under CT + T exposure), while *S. saccharatum* displayed a comparatively more moderate response, suggesting species-specific tolerance mechanisms. This trend is consistent with previous studies indicating that dicotyledonous plants are generally more sensitive to antibiotic contamination due to differences in root structure, permeability, and metabolic pathways [41]. Among the examined endpoints, root elongation was consistently the most sensitive, followed by shoot growth, while germination was less affected, supporting the notion that roots are the primary exposure interface and are highly vulnerable to compounds that inhibit cell division and elongation processes [57]. The enhanced toxicity observed under mixture exposure, particularly for CT + T, indicates additive interactions [58] but also suggests that cumulative effects such as increased intracellular antibiotic accumulation and oxidative stress may amplify phytotoxic responses. Mechanistically, tetracycline toxicity in plants can be attributed to the inhibition of protein synthesis in mitochondria and chloroplasts, induction of oxidative stress through reactive oxygen species (ROS), and chelation of essential nutrients (e.g., Ca^2+^, Mg^2+^), ultimately disrupting physiological processes and growth. These findings are environmentally relevant, as TCs are frequently introduced into agricultural soils through manure, biosolids, and reclaimed wastewater, where they persist due to strong sorption and may be taken up by crops, affecting both plant health and food safety [2,59] (Figure 9).

The EC_50_ values calculated for the experimental models of *D. magna*, *A. salina*, and *A. fischeri* were compared to similar values found in the bibliography. In the cases of 24 h exposure experiments on T, for *Daphnia*, the relevant literature supported that T was not acutely toxic to the organism (EC_50_ > 1000 mg/L) [60,61], consistent with the present study (EC_50_ = 1639 mg/L). OT and T were also not acutely toxic to *Daphnia* after 24 h exposure, with no EC_50_ calculated up to 1000 mg/L under nominal exposure [62]. It is noted that other endpoints, such as reproduction endpoints or chronic exposures, do produce adverse effects at lower concentrations [62]. *A. salina* was indeed more sensitive than *D. magna* after 24 h exposure, as shown by the values recorded for T [63], which were higher than ours (6.3 mM versus the present value of 1.02 mM). *A. fischeri* was consistently more sensitive to TCs than the crustaceans, with recorded values of 0.16 mM (for 15 min) for T [61] and 0.05 mM and 0.013 mM for OT and CT, respectively [64], which were somewhat lower than the ones recorded here. Nevertheless, it was again verified that CT was more toxic than OT.

However, comparisons with literature-derived EC_50_ values should be made cautiously since differences in exposure time, biological endpoint, assay design, and test matrix can substantially influence toxicity estimates. *Daphnia magna* toxicity data are commonly based on 24 or 48 h immobilization tests in freshwater systems, whereas *Artemia salina* assays are conducted in saline media, and *Aliivibrio fischeri* responses are typically measured as short-term inhibition of bioluminescence after minutes rather than hours of exposure. Moreover, reported concentrations may be expressed either in mg/L or mM, and these units are not directly comparable without molecular-weight conversion. Environmental matrix properties, including pH, ionic strength, and sorption behavior, may further alter tetracycline bioavailability. Therefore, the literature comparisons presented here for *D. magna* [60,62], *A. salina* [63], and *A. fischeri,* [46,47,64] should be considered indicative and contextual rather than strictly equivalent.

The binary (CT + T, OT + T) and ternary mixtures revealed toxicity that did not significantly deviate from concentration addition, which is usually the norm for mixtures with similar action [58]. However, the steep toxicity increase observed for *A. fischeri* suggests that synergistic interactions may also occur. Such behavior has previously been associated with multiple compound action on the bacterium, resulting in greater-than-additive inhibitory effects [64]. Recent evidence indicates that joint toxicity in bacterial assays may produce stimulating, adaptive responses or competition for similar intracellular targets at low doses, but this may become synergism in the inhibitory dose range [65]. This mode of action (antagonism at low doses and synergism at higher doses) has also been verified in the *Chlorella pyrenoidosa* model [66]. In general, interactions may include formation of complexes facilitating antagonism or, in some cases, synergism [67]. They may also include enhanced production of reactive oxygen species (ROS) in synergistic conditions [68] and changes in membrane permeability that affects the bioavailability of the joint pollutants [69], among others. According to [68], the dose-dependent deviations from CA are difficult to explain using a particular mechanism without more in-depth mechanistic studies.

Especially for antibiotic mixtures, these combinations can exert greater toxic effects than those expected from individual compounds. Experimental studies with luminescent bacteria have demonstrated that mixture toxicity is frequently consistent with concentration addition, while combinations may also exhibit synergistic interactions that further enhance toxicity [70,71]. Studies on environmental matrices have shown that binary and multi-component antibiotic mixtures often display non-additive behavior; especially for TCs, synergism between them and macrolides or quinolones is possible for aquatic organisms. In the same research, no safe prognosis could be made for TC-TC interactions [72]. However, it has been shown that combined OT and T exposure caused a significant performance reduction in anaerobic digesters that utilize bacteria [11,73]. These findings highlight that mixture effects represent a critical but often underestimated component of environmental risk, emphasizing the need to move beyond single-compound assessments toward more integrative approaches that better reflect real-world contamination patterns. The deviation from additivity towards higher-than-additive toxic responses for *A. fischeri* is of particular concern because it underscores the underestimation of the risk of microbial activity perturbation in ecosystems, where multiple antibiotic stressors are present. Despite providing valuable insights into tetracycline toxicity, this study has certain limitations. The experiments were conducted under controlled laboratory conditions using concentrations higher than those typically observed in the environment, which may limit direct environmental extrapolation. Furthermore, bioaccumulation processes and chronic exposure scenarios were not assessed, although they are known to play a significant role in antibiotic fate and effects. Future research should focus on long-term exposure, environmentally relevant concentrations, and the integration of bioaccumulation and trophic transfer processes.

### 4.2. Integration of a Bioassay Battery for Ecotoxicological Responses

**Figure 10:**
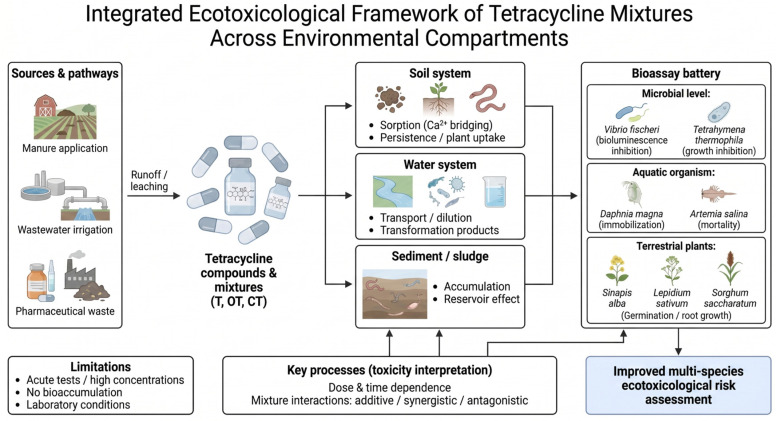
Conceptual framework illustrating the pathways, environmental compartments, and multi-trophic ecotoxicological assessment of tetracycline compounds and their mixtures. The applied battery of bioassays integrates microbial, aquatic, and terrestrial responses, enabling a cross-compartment evaluation of toxicity and mixture interactions.

The present study was designed to move beyond single-species ecotoxicological assessments by integrating a battery of bioassays representing distinct trophic levels and environmental compartments, as illustrated in Figure 10 (Integrated Ecotoxicological Framework). This approach enables a system-level interpretation of tetracycline (TC) toxicity, linking microbial, aquatic, and terrestrial responses under a unified experimental design.

The selected organisms represent key ecological functions: microbial systems (*A. fischeri*, *T. thermophila*) reflect biochemical and cellular-level responses, crustaceans (*D. magna*, *A. salina*) represent aquatic primary consumers, and plants (*S. alba*, *L. sativum*, *S. saccharatum*) represent terrestrial primary producers. By combining these models, the study captures the propagation of toxic effects across trophic levels and environmental compartments, providing a more realistic representation of environmental exposure scenarios.

A critical aspect of this integration is the consistent application of statistical modeling across all experimental systems. Dose–response relationships were systematically analyzed using generalized linear models, regression analysis, and probit analysis for EC_50_ estimation, ensuring comparability of results across species. Despite differences in biological endpoints, all systems demonstrated coherent concentration–response trends, reinforcing the robustness of the experimental design. The use of log-transformed concentrations and standardized endpoints further allowed cross-comparison of sensitivity among organisms, revealing consistent patterns such as the higher responsiveness of microbial systems and *Artemia salina*.

Importantly, the evaluation of mixture toxicity through the concentration addition (CA) model provides a unifying framework linking all bioassays. The observation that most mixtures followed additive behavior, with deviations in specific cases, suggests that TCs share similar modes of action while still allowing for interaction effects under certain biological conditions. These findings highlight the interconnected nature of toxicity across compartments, where microbial-level responses may amplify or precede higher trophic effects.

In addition, the findings contribute to the broader One Health framework, emphasizing the interconnectedness between environmental contamination, ecosystem health, and potential risks to human well-being through food chains and water resources.

Overall, the integration of multiple bioassays through a common statistical and conceptual framework represents a significant advancement over conventional single-endpoint studies. This approach not only enhances the ecological relevance of the findings but also supports the development of more comprehensive and sustainable environmental risk assessment strategies, particularly in the context of emerging contaminants such as antibiotics.

## 5. Conclusions

This study showed that T, OT, and CT all caused concentration-dependent toxicity, but their relative toxicity differed among organisms. Overall, *A. fischeri* and *A. salina* were the most sensitive bioassays, *D. magna* was less sensitive, and dicot plants were more responsive than the monocot species. CT and OT generally exerted stronger effects than T, although no single toxicity ranking applied across all endpoints. A limitation of the study is that it was based on acute laboratory exposures at concentrations above typical ambient levels and did not address chronic toxicity, bioaccumulation, or analytically verified transformation during exposure. Future studies should focus on environmentally realistic exposures, chronic and mixture toxicity, transformation products, and cross-compartment ecological effects.

## Figures and Tables

**Table 1:** Main statistical indices for the *A. fischeri* bioassay (5, 15 and 30 min). Key: N/S, not significant; * *p* < 0.05, ** *p* < 0.01.

Exp System	Mauchly’s Test	Time on Inhibition	Dose on Inhibition	Time × Dose Interaction
CT	0.121	F(2): 136.57, *p* < 0.01, η^2^ = 0.91 **	F(5):29.54, *p* < 0.01, η^2^ = 0.99 **	F(10):9.52, *p* < 0.01, η^2^ = 0.79 **
T	0.077	F(2): 221.11, *p* < 0.01, η^2^ = 0.95 **	F(4):4.26, *p* < 0.05, η^2^ = 0.63 *	F(8):1.14, *p* > 0.05 N/S
OT	0.02 * Greenhouse–Geisser values used	F(2): 38.11, *p* < 0.01, η^2^ = 0.79 **	F(4):23.29, *p* < 0.01, η^2^ = 0.90 **	F(8):2.77, *p* > 0.05 N/S
CT + T	0.663	F(2) = 72.84, *p* < 0.001, η^2^ = 0.901 **	F(3) = 8.311, *p* < 0.05, η^2^ = 0.757 *	F(6) = 4.13, *p* < 0.05, η^2^ = 0.608 *
OT + T	0.148	F(2) = 9.94, *p* < 0.01, η^2^ = 0.554 **	F(3) = 11.95, *p* < 0.01, η^2^ = 0.81 **	F(6) = 0.16, *p* > 0.05 N/S
CT + T + OT	0.145	F(2) = 10.95, *p* < 0.01, η^2^ = 0.804 **	F(3) = 11.95, *p* < 0.01, η^2^ = 0.810 **	F(6) = 0.15, *p* > 0.05 N/S

**Table 2:** Main statistical indices for *D. magna* (**A**) and *A. salina* (**B**) bioassay. Key: ** good; * adequate.

***Daphnia magna (A)***					
Exp system	Model fit	B estimate	95% CI	Wald χ^2^	*p*-value
CT	deviance/df = 1.11; Pearson χ^2^/df = 0.89 **	1.597	0.572–2.623	9.319	=0.002
T	deviance/df = 1.12; Pearson χ^2^/df = 0.91 **	1.95	0.882–3.034	12.71	<0.001
OT	deviance/df = 1.23; Pearson χ^2^/df = 1.12 *	3.69	2.287–5.104	26.43	<0.001
CT + T	deviance/df = 0.92; Pearson χ^2^/df = 0.81 **	2.58	0.993–4.168	10.15	<0.01
OT + T	deviance/df = 1.13; Pearson χ^2^/df = 1.23 *	7.19	4.305–10.085	23.80	<0.001
CT + T + OT	deviance/df = 1.13; Pearson χ^2^/df = 1.08 *	5.05	2.896–7.204	21.18	<0.001
***Artemia salina (B)***					
CT	deviance/df = 1.72; Pearson χ^2^/df = 1.62 *	2.91	1.60–4.23	18.92	<0.001
T	deviance/df = 0.79; Pearson χ^2^/df = 0.67 **	3.83	2.37–5.30	26.41	<0.001
OT	deviance/df = 0.67; Pearson χ^2^/df = 0.62 **	4.83	2.88–6.79	23.51	<0.001
CT + T	deviance/df = 1.12; Pearson χ^2^/df = 1.04 *	5.11	2.95–7.27	21.52	<0.001
OT + T	deviance/df = 0.60; Pearson χ^2^/df = 0.70 **	4.68	2.41–6.58	16.30	<0.001
CT + T + OT	deviance/df = 0.73; Pearson χ^2^/df = 0.71 **	3.96	1.91–6.00	14.35	<0.001

**Table 3:** Single, binary and tertiary mixture results on *A. fischeri*, *D. magna*, *A. salina*, *S. saccharatum*.

Exp System	Mixture Ratio	EC_50_ (mM)	Possible Mechanism/Effect
***A. fischeri***			
CT	-	0.91	
T	-	1.13	
OT	-	1.44	
CT + T	1:1	0.16 (1.02)	enhanced toxicity
OT + T	7:3	0.73 (1.11)	possible additivity
CT + T + OT	3.5:3.5:3	0.26 (1.10)	enhanced toxicity
***D. magna***			
CT	-	5.54	
T	-	3.69	
OT	-	3.39	
CT + T	1:1	10.1 (4.44)	reduced toxicity
OT + T	7:3	5.08 (3.48)	possible additivity
CT + T + OT	3.5:3.5:3	2.54 (4.08)	possible additivity
***A. salina***			
CT	-	0.88	
T	-	1.02	
OT	-	0.8	
CT + T	1:1	3.39 (0.94)	reduced toxicity
OT + T	7:3	5.08 (0.85)	reduced toxicity
CT + T + OT	3.5:3.5:3	2.54 (0.89)	reduced toxicity
***Sorghum saccharatum:*** ***Seed germination inhibition***			
CT	-	3.36	
T	-	4.88	
OT	-	1.62	
CT + T	1:1	2.71 (4.00)	possible additivity
OT + T	7:3	3.06 (2.03)	possible additivity

Note: Values in brackets are the ones derived from the CA formula.

## Data Availability

The original contributions presented in this study are included in the article/Supplementary Material. Further inquiries can be directed to the corresponding authors.

## Supplementary Materials

The following supporting information can be downloaded at: https://www.mdpi.com/article/10.3390/jox16040122/s1. Table S1: EC50/IC50 values.

## Abbreviations

The following abbreviations are used in this manuscript:

CA	Concentration addition
CT	Chlorotetracycline
EC_50_	Effective concentration 50%
OT	Oxytetracycline
SDGs	Sustainable Development Goals
TCs	Tetracyclines
