# Supplementary material for: A Comparative Multi-Bioassay Assessment of Tetracycline Mixture Toxicity in Water and Soil Using Harmonized Dose–Response Modeling
Source: J Xenobiot. 2026 Jul 1;16(4):122. doi: 10.3390/jox16040122 (PMC13397924; doi:10.3390/jox16040122)
Supplement: Supplementary file 1 [file jox-16-00122-s001.zip › jox-4366634-supplementary.pdf]

Supplementary Materials: A Comparative Multi-Bioassay Assessment of Tetracycline Mixture Toxicity in Water and Soil Using Harmonized Dose–Response Modeling

Chrysi A. Papadimitriou, Christina Emmanouil, Amalia Moriki and Vasileios Bartzis

Table S1:EC50/IC50 values

plants CT	dead		
CONC	sinapis	lepidium	sorgum
1.33	0	0	0
1.33	0	0	0
1.33	0	0	0
MEAN	0.000	0.000	0.000
SDEV	0	0	0
SE	0	0	0
1.63	0	0	1
1.63	0	0	1
1.63	0	0	1
MEAN	0.00	0.00	1.00
SDEV	0	0	0
SE	0	0	0
1.93	2	1	3
1.93	2	1	3
1.93	2	1	2
MEAN	2	1	2.666667
SDEV	0	0	0.57735
SE	0	0	0.333333
2.23	3	1	5
2.23	3	2	5
2.23	3	3	4
MEAN	3	3	4
SDEV	0	1	0.57735
SE	0	0.57735	0.333333
2.53	4	4	6
2.53	5	4	6
2.53	4	3	6
MEAN	4.333333	3.666667	6
SDEV	0.57735	0.57735	0
SE	0.333333	0.333333	0

plants 3	dead		
CONC	sinapis	lepidium	sorgum
1.89	5	6	10
1.89	5	5	10
1.89	4	6	9
MEAN	4.666667	5.666667	9.66666667
SDEV	0.57735	0.57735	0.57735027
SE	0.333333	0.333333	0.33333333
2.19	9	10	10
2.19	10	9	9
2.19	10	10	10
MEAN	9.666667	9.666667	9.66666667
SDEV	0.57735	0.57735	0.57735027
SE	0.333333	0.333333	0.33333333
2.49	10	10	10
2.49	10	10	10
2.49	10	9	10
MEAN	10	9.666667	10
SDEV	0	0.57735	0
SE	0	0.333333	0

tet CT	% inh	tet T	% inh	tet OT	% inh
CONC		CONC		CONC	
1.33	0	1.33	0	1.59	0
1.33	0	1.33	0	1.59	0
1.33	0	1.33	0	1.59	0
MEAN	0.000	MEAN	0.000	MEAN	0
SDEV	0	SDEV	0	SDEV	0
SE	0	SE	0	SE	0
1.63	11	1.53	10	1.89	28
1.63	23	1.53	22	1.89	35
1.63	13	1.53	28	1.89	33
MEAN	15.67	MEAN	20.00	MEAN	32
SDEV	6.429101	SDEV	9.165151	SDEV	3.605551
SE	3.711843	SE	5.291503	SE	2.081666
1.93	24	1.93	25	2.19	38
1.93	36	1.93	43	2.19	46
1.93	60	1.93	52	2.19	50
MEAN	40	MEAN	40	MEAN	44.66667
SDEV	18.3303	SDEV	13.74773	SDEV	6.110101
SE	10.58301	SE	7.937254	SE	3.527668
2.23	44	2.23	42	2.49	50
2.23	51	2.23	64	2.49	59
2.23	55	2.23	53	2.49	58
MEAN	13	MEAN	53	MEAN	55.66667
SDEV	6.429101	SDEV	11	SDEV	4.932883
SE	3.711843	SE	6.350853	SE	2.848001
2.53	40	2.53	60		
2.53	60	2.53	79		
2.53	82	2.53	44		
MEAN	60.66667	MEAN	61		
SDEV	21.00794	SDEV	17.52142		
SE	12.12894	SE	10.11599		

tet CT+T	% inh	tet OT+T	% inh	tet 3	% inh
CONC		CONC		CONC	
1.93	0	1.93	0	1.89	3
1.93	0	1.93	0	1.89	2
1.93	0	1.93	0	1.89	2
MEAN	0	MEAN	0	MEAN	2.333333
SDEV	0	SDEV	0	SDEV	0.57735
SE	0	SE	0	SE	0.333333
2.23	33	2.23	36	2.19	2
2.23	42	2.23	45	2.19	4
2.23	39	2.23	54	2.19	3
MEAN	38	MEAN	45	MEAN	3
SDEV	4.582576	SDEV	9	SDEV	1
SE	2.645751	SE	5.196152	SE	0.57735
2.53	49	2.53	51	2.49	5
2.53	66	2.53	50	2.49	5
2.53	50	2.53	49	2.49	4
MEAN	55	MEAN	50	MEAN	4.666667
SDEV	9.539392	SDEV	1	SDEV	0.57735
SE	5.507571	SE	0.57735	SE	0.333333
				2.8	5
				2.8	5
				2.8	5
				MEAN	5
				SDEV	0
				SE	0

artemia CT	dead	artemia T	dead	artemia OT	dead
CONC		CONC		CONC	
1.33	2	1.33	1	1.59	1
1.33	2	1.33	1	1.59	2
1.33	1	1.33	1	1.59	1
1.33	1	1.33	0	1.59	0
MEAN	1.500	MEAN	0.750	MEAN	1
SDEV	0.57735	SDEV	0.5	SDEV	0.816497
SE	0.333333	SE	0.288675	SE	0.471405

1.63	3	1.63	3	1.89	4
1.63	3	1.63	2	1.89	3
1.63	3	1.63	2	1.89	3
1.63	1	1.63	1	1.89	2
MEAN	2.50	MEAN	2.00	MEAN	3
SDEV	1	SDEV	0.816497	SDEV	0.816497
SE	0.57735	SE	0.471405	SE	0.471405
1.93	1	1.93	3	2.19	5
1.93	5	1.93	3	2.19	4
1.93	5	1.93	3	2.19	4
1.93	5	1.93	1	2.19	3
MEAN	1	MEAN	2.5	MEAN	4
SDEV	2	SDEV	1	SDEV	0.816497
SE	1.154701	SE	0.57735	SE	0.471405
2.23	5	2.23	5	2.49	5
2.23	5	2.23	5	2.49	5
2.23	4	2.23	4	2.49	4
2.23	2	2.23	4	2.49	5
MEAN	4	MEAN	4.5	MEAN	4.75
SDEV	1.414214	SDEV	0.57735	SDEV	0.5
SE	0.816497	SE	0.333333	SE	0.288675
2.53	5	2.53	5	2.8	5
2.53	5	2.53	5	2.8	5
2.53	5	2.53	4	2.8	5
2.53	4	2.53	5	2.8	5
MEAN	4.75	MEAN	4.75	MEAN	5
SDEV	0.5	SDEV	0.5	SDEV	0
SE	0.288675	SE	0.288675	SE	0

artemia CT+T	dead	artemia OT+T	dead	artemia 3	dead
CONC		CONC		CONC	
1.89	1	1.89	2	1.89	3
1.89	1	1.89	2	1.89	2
1.89	0	1.89	1	1.89	2
1.89	1	1.89	2	1.89	1
MEAN	0.75	MEAN	1.75	MEAN	2
SDEV	0.5	SDEV	0.5	SDEV	0.816497
SE	0.28867	SE	0.28867	SE	0.47140

	5		5		5
2.19	3	2.19	3	2.19	2
2.19	3	2.19	3	2.19	4
2.19	3	2.19	3	2.19	3
2.19	1	2.19	4	2.19	4
MEAN	2.5	MEAN	3.25	MEAN	3.25
SDEV	1	SDEV	0.5	SDEV	0.95742
					7
SE	0.57735	SE	0.28867	SE	0.55277
			5		1
2.49	5	2.49	5	2.49	5
2.49	5	2.49	5	2.49	5
2.49	4	2.49	4	2.49	4
2.49	2	2.49	5	2.49	4
MEAN	4	MEAN	4.75	MEAN	4.5
SDEV	1.41421	SDEV	0.5	SDEV	0.57735
	4				
SE	0.81649	SE	0.28867	SE	0.33333
	7		5		3
2.8	5	2.8	5	2.8	5
2.8	5	2.8	5	2.8	5
2.8	4	2.8	4	2.8	5
2.8	5	2.8	5	2.8	4
MEAN	4.75	MEAN	4.75	MEAN	4.75
SDEV	0.5	SDEV	0.5	SDEV	0.5
SE	0.28867	SE	0.28867	SE	0.28867
	5		5		5

dapnia CT	dead	daphnia T	dead	daphnia OT	dead
CONC		CONC		CONC	
1.33	2	1.33	1	1.59	2
1.33	1	1.33	1	1.59	1
1.33	1	1.33	1	1.59	0
1.33	0	1.33	0	1.59	0
MEAN	1.000	MEAN	0.750	MEAN	0.75
SDEV	0.81649	SDEV	0.5	SDEV	0.95742
	7				7
SE	0.47140	SE	0.28867	SE	0.55277
	5		5		1
1.63	3	1.63	2	1.89	2
1.63	2	1.63	2	1.89	2
1.63	2	1.63	1	1.89	1
1.63	1	1.63	1	1.89	1
MEAN	2.00	MEAN	1.50	MEAN	1.5
SDEV	0.81649	SDEV	0.57735	SDEV	0.57735
	7				

SE	0.47140	SE	0.33333	SE	0.33333
	5		3		3
1.93	2	1.93	3	2.19	4
1.93	2	1.93	2	2.19	2
1.93	2	1.93	2	2.19	3
1.93	1	1.93	1	2.19	1
MEAN	1	MEAN	2	MEAN	2.5
SDEV	0.5	SDEV	0.81649	SDEV	1.29099
			7		4
SE	0.28867	SE	0.47140	SE	0.74535
	5		5		6
2.23	3	2.23	4	2.49	5
2.23	3	2.23	3	2.49	5
2.23	2	2.23	2	2.49	4
2.23	2	2.23	1	2.49	2
MEAN	2	MEAN	2.5	MEAN	4
SDEV	0.57735	SDEV	1.29099	SDEV	1.41421
			4		4
SE	0.33333	SE	0.74535	SE	0.81649
	3		6		7
2.53	5	2.53	5	2.8	5
2.53	5	2.53	5	2.8	5
2.53	2	2.53	2	2.8	4
2.53	2	2.53	2	2.8	5
MEAN	3.5	MEAN	3.5	MEAN	4.75
SDEV	1.73205	SDEV	1.73205	SDEV	0.5
	1		1		
SE	1	SE	1	SE	0.28867
					5

daphnia CT+T	dead	daphnia OT+T	dead	daphnia 3	dead
CONC		CONC		CONC	
1.89	2	1.89	0	1.89	1
1.89	1	1.89	0	1.89	1
1.89	0	1.89	0	1.89	1
1.89	0	1.89	0	1.89	0
MEAN	0.75	MEAN	0	MEAN	0.75
SDEV	0.95742	SDEV	0	SDEV	0.5
	7				
SE	0.55277	SE	0	SE	0.28867
	1				5
2.19	1	2.19	3	2.19	2
2.19	1	2.19	2	2.19	2
2.19	0	2.19	2	2.19	3
2.19	2	2.19	1	2.19	4
MEAN	1	MEAN	2	MEAN	2.75
SDEV	0.81649	SDEV	0.81649	SDEV	0.95742

	7		7		7
SE	0.47140<br>5	SE	0.47140<br>5	SE	0.55277<br>1
2.49	2	2.49	5	2.49	5
2.49	2	2.49	5	2.49	4
2.49	2	2.49	4	2.49	5
2.49	3	2.49	2	2.49	2
MEAN	2.25	MEAN	4	MEAN	4
SDEV	0.5	SDEV	1.41421<br>4	SDEV	1.41421<br>4
SE	0.28867<br>5	SE	0.81649<br>7	SE	0.81649<br>7
2.8	2	2.8	5	2.8	5
2.8	2	2.8	5	2.8	5
2.8	4	2.8	4	2.8	4
2.8	4	2.8	5	2.8	5
MEAN	3	MEAN	4.75	MEAN	4.75
SDEV	1.15470<br>1	SDEV	0.5	SDEV	0.5
SE	0.66666<br>7	SE	0.28867<br>5	SE	0.28867<br>5

VIBRIO CT	INH%		
CONC	5 MIN	15 MIN	30 MIN
12.5	25	20	47
12.5	25	25	35
12.5	37	37	63
MEAN	29.000	27.33333	48.3333<br>3
SDEV	6.928203	8.736895	14.0475<br>4
SE	4	5.044249	8.11035
25	32	32	66
25	35	35	72
25	41	41	80
MEAN	36.00	36.000	72.6666<br>7
SDEV	4.582576	4.582576	7.02376<br>9
SE	2.645751	2.645751	4.05517<br>5
50	50	50	66
50	60	60	81
50	66	66	72
MEAN	58.66667	58.66667	73
SDEV	8.082904	8.082904	7.54983

VIBRIO T	INH%		
CONC	5 MIN	15 MIN	30 MIN
12.5	25	54	50
12.5	29	62	59
12.5	33	69	71
MEAN	29.000	61.667	60.000
SDEV	4	7.50555<br>3	10.5356<br>5
SE	2.30940<br>1	4.33333<br>3	6.08276<br>3
25	35	68	70
25	31	58	61
25	39	48	75
MEAN	35.00	58.00	68.67
SDEV	4	10	7.09459<br>9
SE	2.30940<br>1	5.77350<br>3	4.09606<br>9
50	39	56	73
50	47	77	83
50	43	67	79
MEAN	43	66.6666<br>7	78.3333<br>3
SDEV	4	10.5039	5.03322

			4
SE	4.666667	4.666667	4.358899
100	69	69	68
100	85	75	85
100	81	81	100
MEAN	78.33333	75	84.33333
SDEV	8.326664	6	16.01041
SE	4.807402	3.464102	9.243616
500	76	76	95
500	88	82	100
500	82	88	100
MEAN	82	82	98.33333
SDEV	6	6	2.886751
SE	3.464102	3.464102	1.666667
1500	82	81	90
1500	90	90	100
1500	98	98	100
MEAN	90	89.66667	96.66667
SDEV	8	8.504901	5.773503
SE	4.618802	4.910307	3.333333

		7	3
SE	2.309401	6.064468	2.905933
75	45	57	79
75	50	75	90
75	55	93	84
MEAN	50	75	84.33333
SDEV	5	18	5.507571
SE	2.886751	10.3923	3.179797
100	43	64	80
100	52	74	84
100	55	84	85
MEAN	50	74	83
SDEV	6.244998	10	2.645751
SE	3.605551	5.773503	1.527525

VIBRIO OT	INH%		
CONC	5 MIN	15 MIN	30 MIN
12.5	8	15	20
12.5	10	11	25
12.5	9	13	29
MEAN	9	13	24.66667
SDEV	1	2	4.50925
SE	0.57735	1.154701	2.603417
25	14	27	30
25	12	19	39
25	16	35	36
MEAN	14	27	35
SDEV	2	8	4.582576

VIBRIO CT+T	INH%		
CONC	5 MIN	15 MIN	30 MIN
1.56	5	13	10
1.56	19	19	21
1.56	13	22	18
MEAN	12.33333	18	16.33333
SDEV	7.023769	4.582576	5.686241
SE	4.055175	2.645751	3.282953
3.12	26	38	37
3.12	32	24	44
3.12	19	49	47
MEAN	25.66667	37	42.66667
SDEV	6.506407	12.52996	5.131601

SE	1.154701	4.618802	2.645751
50	34	24	34
50	42	40	46
50	38	56	50
MEAN	38	40	43.33333
SDEV	4	16	8.326664
SE	2.309401	9.237604	4.807402
75	56	77	63
75	51	59	47
75	46	41	73
MEAN	51	59	61
SDEV	5	18	13.11488
SE	2.886751	10.3923	7.571878
100	54	80	84
100	66	69	71
100	60	92	97
MEAN	60	80.33333	84
SDEV	6	11.50362	13
SE	3.464102	6.64162	7.505553

SE	3.756476	7.234178	2.962731
6.25	30	40	45
6.25	40	60	54
6.25	39	50	65
MEAN	36.33333	50	54.66667
SDEV	5.507571	10	10.01665
SE	3.179797	5.773503	5.783117
12.5	26	34	45
12.5	40	64	56
12.5	49	50	69
MEAN	38.33333	49.33333	56.66667
SDEV	11.59023	15.01111	12.01388
SE	6.69162	8.666667	6.936217

VIBRIO OT+T	INH%		
CONC	5 MIN	15 MIN	30 MIN
1.56	44	56	64
1.56	47	40	59
1.56	35	66	75
MEAN	42	54	66
SDEV	6.244998	13.11488	8.185353
SE	3.605551	7.571878	4.725816
3.12	55	62	50
3.12	48	64	66
3.12	38	57	81
MEAN	47	61	65.66667
SDEV	8.544004	3.605551	15.50269
SE	4.932883	2.081666	8.950481

VIBRIO 3	INH%		
CONC	5 MIN	15 MIN	30 MIN
1.56	32	46	49
1.56	20	32	36
1.56	46	60	65
MEAN	32.66667	46	50
SDEV	13.01281	14	14.52584
SE	7.512952	8.082904	8.386497
3.12	30	44	33
3.12	43	48	57
3.12	52	55	75
MEAN	41.66667	49	55
SDEV	11.06044	5.567764	21.07131
SE	6.385748	3.21455	12.16553

6.25	60	55	56
6.25	53	65	73
6.25	39	76	81
MEAN	50.66667	65.33333	70
SDEV	10.69268	10.50397	12.76715
SE	6.17342	6.064468	7.371115
12.5	68	77	70
12.5	63	73	77
12.5	53	74	80
MEAN	61.33333	74.66667	75.66667
SDEV	7.637626	2.081666	5.131601
SE	4.409586	1.20185	2.962731

6.25	69	66	64
6.25	67	73	83
6.25	68	86	78
MEAN	68	80	75
SDEV	1	20	9.848858
SE	0.57735	11.54701	5.686241
12.5	100	100	100
12.5	80	92	100
12.5	60	81	95
MEAN	80	91	98.33333
SDEV	20	9.539392	2.886751
SE	11.54701	5.507571	1.666667
